# Supplementary material for: Psychological Intervention for Patients with Biopsychosocial Late Effects Following Surgery for Colorectal Cancer with Peritoneal Metastases—A Feasibility Study
Source: Cancers (Basel). 2025 Mar 27;17(7):1127. doi: 10.3390/cancers17071127 (PMC11987789; doi:10.3390/cancers17071127)
Supplement: Supplementary file 1 [file cancers-17-01127-s001.zip › Supplemental material S1.pdf]

Supplementary material S1: Patient characteristics

	**Patients participating in the MDT* n = 13**	**Patients not participating in the MDT n = 15**
**Gender**		
Female	11 (85%)	4 (27%)
Male	2 (15%)	11 (73%)
**Age, years (mean, range)**	59 (39-72)	66 (53-79)
**Prior psychiatric disease****		
Yes	4 (31%)	
No	9 (69%)	NA

Proportions are presented as percentages.

* MDT: Multidisciplinary team

** Prior psychiatric disease: Having received a psychiatric diagnosis at any time during their lifetime.
