# Supplementary figures and images for: Psychological Intervention for Patients with Biopsychosocial Late Effects Following Surgery for Colorectal Cancer with Peritoneal Metastases—A Feasibility Study
Source: Cancers (Basel). 2025 Mar 27;17(7):1127. doi: 10.3390/cancers17071127 (PMC11987789; doi:10.3390/cancers17071127)

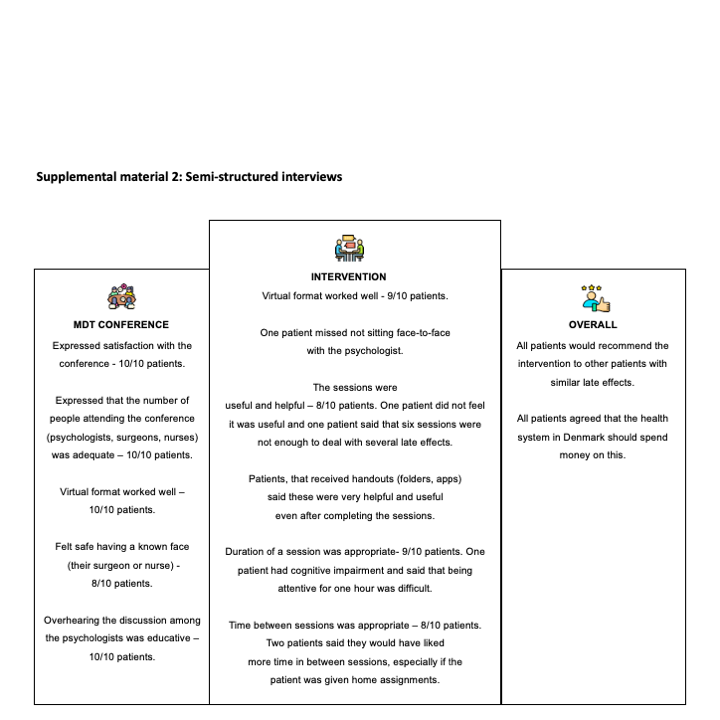

Supplement: Supplementary file 1 [file cancers-17-01127-s001.zip › Supplemental material S2 Semistructured interviews.tiff]
